# The Impact of Various Reactive Oxygen Species on the Formation of Neutrophil Extracellular Traps 

**DOI:** 10.1155/2012/849136

**Published:** 2012-01-26

**Authors:** Tina Kirchner, Sonja Möller, Matthias Klinger, Werner Solbach, Tamás Laskay, Martina Behnen

**Affiliations:** ^1^Institute for Medical Microbiology and Hygiene, University of Lübeck, Ratzeburger Allee 160, 23538 Lübeck, Germany; ^2^Institute of Anatomy, University of Lübeck, Ratzeburger Allee 160, 23538 Lübeck, Germany

## Abstract

The formation of neutrophil extracellular traps (NETs) depends on the generation of reactive oxygen species (ROS). Previous studies revealed that both NADPH oxidase and myeloperoxidase (MPO) are required for NET release. However, the contribution of various ROS as well as the role of mitochondria-derived ROS has not been addressed so far. In the present study we aimed to investigate in a systematic and comprehensive manner the contribution of various ROS and ROS-generating pathways to the PMA-induced NET release. By using specific inhibitors, the role of both NADPH oxidase- and mitochondria-derived ROS as well as the contribution of superoxide dismutase (SOD) and MPO on the NET release was assessed. We could demonstrate that NADPH oxidase function is crucial for the formation of NETs. In addition, we could clearly show the involvement of MPO-derived ROS in NET release. Our results, however, did not provide evidence for the role of SOD- or mitochondria-derived ROS in NET formation.

## 1. Introduction

In addition to the well-known capacity of neutrophils to phagocytose and kill invading microorganisms intracellularly [1], neutrophils can capture and kill pathogens extracellularly through the release of neutrophil extracellular traps (NETs) [2]. These complex three-dimensional structures contain several antimicrobial neutrophil granule proteins which are attached to a DNA backbone [2]. The novel cell death mechanism NETosis has been described as the mechanism leading to the formation of NETs [3, 4]. Studies with neutrophils from patients with chronic granulomatous disease (CGD) indicated an essential role of NADPH oxidase activity in PMA-induced NET release [5]. Although the oxidant dependence of PMA-induced NET formation has been established, no comprehensive studies have been carried out so far to assess the role of individual reactive oxygen species (ROS) and/or the enzymatic pathways involved in their generation.

Patients completely deficient in myeloperoxidase (MPO) fail to form neutrophil extracellular traps upon exposure to PMA [6]. A regulatory role of MPO on the NET release has also been described [7]. The observation that singlet oxygen is essential for NET formation [8] further substantiates the involvement of MPO and MPO-derived hypochlorous acid (HOCl) in NET formation.

In addition to NADPH oxidase, the mitochondrial electron transport chain is another source of intracellular ROS. The contribution of mitochondria-derived ROS regarding its contribution to NET formation, however, has not been addressed so far.

In the present study we aimed to investigate in a systematic and comprehensive manner the contribution of various reactive oxygen species and ROS-generating pathways to the PMA-induced NET release. By using specific inhibitors, the impact of both the NADPH- and mitochondria-derived ROS as well as the contribution of superoxide dismutase (SOD) and myeloperoxidase (MPO) on the NET release was assessed. The results confirm previous findings that NADPH oxidase function is crucial for the formation of NETs. In addition, we could clearly show the involvement of oxidative MPO functions in NET release. However, according to our results, neither the mitochondria-derived ROS nor SOD play a major role in NET formation.

## 2. Materials and Methods

### 2.1. Isolation and Culture of Primary Human Neutrophils

Peripheral blood was collected by venipuncture from healthy adult volunteers using lithium heparin. Neutrophils were isolated as described previously [9]. The blood collection was conducted with the understanding and the consent of each participant and was approved by the ethical committee of the Medical Faculty of the University of Lübeck (05-124). The cell preparations contained >99.9% granulocytes as determined by morphological examination of Giemsa-stained cytocentrifuged slides (Shandon, Pittsburgh, PA) [10]. Neutrophils were cultured using complete medium (RPMI 1640 medium supplemented with 50 *μ*M 2-mercaptoethanol, 10 mM HEPES, 10% heat inactivated fetal bovine serum (all from Sigma-Aldrich, Steinheim, Germany), 4 mM L-glutamine, 100 U/mL penicillin, 100 *μ*g/mL streptomycin (all from Biochrom, Berlin, Germany)) at 37°C in a humidified air atmosphere containing 5% CO_2_.

### 2.2. Inhibitors

**Figure 1:**
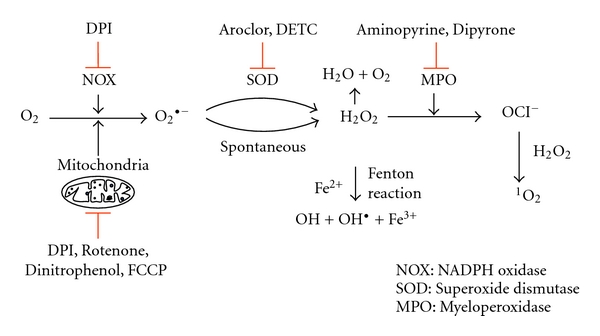
Generation and inhibition of ROS in neutrophils. The generation of superoxide (O_2_^∙−^) from oxygen (O_2_) is mediated either by the NADPH oxidase complex (NOX) or in mitochondria by cytochrome c peroxidase or xanthine oxidase. Superoxide will be converted to hydrogen peroxide (H_2_O_2_) either spontaneously or mediated by superoxide dismutase (SOD). Hydrogen peroxide is a source of hydroxyl radical (OH^•^) via the Fenton reaction. Myeloperoxidase uses hydrogen peroxide as substrate for the formation of halogenated ROS such as hypochlorite (OCl^−^). Reaction of hypochlorite with hydrogen peroxide will result in the formation of singlet oxygen (^1^O_2_). The effect of the inhibitors used in the study (10 *μ*M Rotenone, 10 *μ*M Dinitrophenol, 500 nM FCCP, 20 *μ*M DPI, 38 *μ*M Aroclor, 10 *μ*M DETC, 200 *μ*M Dipyrone and 200 *μ*M Aminopyrine) is indicated.

To inhibit the NADPH oxidase (NOX) diphenyleneiodonium chloride (20 *μ*M, DPI, Sigma-Aldrich, Steinheim, Germany) was used. Inhibitors of myeloperoxidase (MPO) were Dipyrone hydrate (200 *μ*M, Dipyrone) or 4-dimethylaminoantipyrine (200 *μ*M, Aminopyrine, both Sigma-Aldrich). Inhibitors of superoxide dismutase (SOD) were diethyldithiocarbamic acid (10 *μ*M, DETC, Alexis, Lörrach, Germany) or Aroclor 1242 (38 *μ*M, Aroclor, Supelco Analytical, Bellefonte, USA). To inhibit ROS production by the mitochondria the electron transport inhibitor Rotenone (10 *μ*M, Calbiochem, Merck, Darmstadt, Germany) and the uncoupling chain reagents 2, 4-Dinitrophenol (10 *μ*M, Dinitrophenol, Supelco Analytical) or carbonyl cyanide p-[trifluoromethoxy]-phenyl-hydrozone (500 nM FCCP, Sigma-Aldrich) were used (Figure 1).

For all experiments, freshly isolated human neutrophils were preincubated for 30 min at 37°C with the inhibitors listed above. As control, neutrophils were incubated in medium alone.

### 2.3. Assessment of Neutrophil Apoptosis and Viability

Freshly isolated human neutrophils (5 × 10^6^/mL in complete medium) were incubated with or without inhibitors for 4 h at 37°C. The solvents DMSO (0.01%, Sigma-Aldrich) and methanol (1%, Th. Geyer, Renningen, Germany) were used as control. The samples were analyzed by staining with Annexin V-FITC (Promokine, Heidelberg, Germany) and propidium iodide (Sigma-Aldrich). Annexin V exhibits calcium-dependent binding to phosphatidylserine (PS) expressed in the outer membrane leaflet of apoptotic PMN [11]. Labeling of apoptotic cells with Annexin V-FITC and counterstaining with propidium iodide (PI) for necrotic cells were performed as recommended by the manufacturers. The labeled cells were analyzed immediately by flow cytometry using a FACS Calibur flow cytometer and the CellQuest Pro software (BD Biosciences, San Diego, USA).

### 2.4. Assays for ROS Production

Three assays were used to measure production of intracellular, extracellular, or intracellular and extracellular reactive oxygen species by human neutrophils, respectively.

#### 2.4.1. Intracellular Oxidative Burst Assay Using Flow Cytometry

The intracellular production of ROS was assayed by flow cytometry using the substrate dihydrorhodamine 123 (DHR 123, Invitrogen, Eugene, USA) that diffuses into the cells and is oxidized by ROS to the fluorescent Rhodamine 123. With this method, it is possible to evaluate the response of individual cells. Freshly isolated human neutrophils (5 × 10^6^/mL in complete medium) were preincubated with the previously described inhibitors for 30 min at 37°C. Subsequently, 2 *μ*M DHR 123 were added, and cells were stimulated with 4 *μ*M phorbol myristate acetate (PMA, Sigma-Aldrich) for 5 min at 37°C. The reaction was stopped on ice, and the fluorescence intensity of the cells was analyzed immediately by flow cytometry using a FACS Calibur flow cytometer and the CellQuest Pro software.

#### 2.4.2. Luminol-Amplified Chemiluminescence Assay

The sum of intra- and extracellular ROS was measured by using a luminol-amplified chemiluminescence assay. Due to the fact that MPO-derived metabolites are responsible for the excitation of luminol (5-amino-2,3-dihydro-1,4-phthalazindione) [12] and because neutrophils release MPO from azurophil granules (degranulation) during their activation, not only intracellular but also extracellular ROS can be detected by this technique.

4 × 10^5^ freshly isolated human neutrophils (2 × 10^6^/mL) in CL medium (a custom-made RPMI-modified medium without phenol red and sodium hydrogen carbonate containing 20 mM HEPES (Biochrom, Berlin, Germany)) were seeded in a flat bottom white 96-well plate (Nunclon Delta Surface, Nunc, Langenselbold, Germany) and preincubated with specific inhibitors for 30 min at 37°C. Subsequently 0.06 mM luminol (Sigma-Aldrich) was added, and cells were stimulated with 20 nM PMA (Sigma-Aldrich). Neutrophils without PMA treatment were used as control. The chemiluminescence resulting from ROS production was analyzed immediately using an infinite 200 reader and the Tecan i-control 1.7 Software (Tecan, Crailsheim, Germany). ROS release was monitored every 2 minutes for a period of 1 h at 37°C.

#### 2.4.3. Lucigenin-Amplified Chemiluminescence Assay

The lucigenin-enhanced chemiluminescence assay was used to study the kinetic of superoxide production by neutrophils. Lucigenin specifically emits light upon reaction with superoxide, but is not being excited by MPO-derived ROS [12, 13]. Furthermore lucigenin detects only extracellular but not intracellular ROS because it cannot penetrate the cell membrane [14]. For the lucigenin assay neutrophils were treated as described above in the luminol assay, but with 0.2 mM lucigenin (Alexis, Loerrach, Germany) instead of luminol.

### 2.5. Induction and Detection of Neutrophil Extracellular Traps

Staining with the non-cell-permeable DNA dye SYTOXgreen (Invitrogen) was used to study the kinetics of NET formation by neutrophils incubated with or without specific inhibitors. Per sample 2 × 10^5^ neutrophils in NET medium (CL medium supplemented with 0.5% human serum albumin (Behring, Marburg, Germany) and 10 mM HEPES buffer (PAA, Pasching, Austria)) were seeded to a cellstar 96-well plate (Greiner Bio-One, Frickenhausen, Germany) and preincubated with the specific inhibitors for 30 min at 37°C. To detect the extracellular DNA of neutrophil extracellular traps, 5 *μ*M SYTOXgreen was added, and the neutrophils were stimulated with 20 nM PMA. The fluorescence of NET-bound SYTOXgreen (excitation: 488 nm, emission: 510 nm) was analyzed for a period of 4 h every 5 min at 37°C using an infinite 200 reader and Tecan i-control 1.7 Software (Tecan).

### 2.6. Microscopical Assessment of NET Formation

To visualize NETs, fluorescence microscopy and scanning electron microscopy (SEM) were performed. For these techniques freshly isolated human neutrophils were centrifuged for 10 min, 1045 ×g, and resuspended in NET medium (1 × 10^6^/mL). 5 × 10^5^ neutrophils with inhibitors or in medium alone were settled on poly-L-lysine-coated coverslips (BD BioCoat Cellware, Bedford, USA) for fluorescence microscopy or on thermanox slides (Thermanox coverslips, Nunc) for SEM. After incubation for 30 min at 37°C NET formation was induced with 20 nM PMA for 4 h. Samples without PMA were used as control. At this point the further treatment for fluorescence and SEM samples differs. For fluorescence microscopy the samples were fixed with 4% paraformaldehyde (Sigma-Aldrich) for 10 min at room temperature. Subsequently, the supernatant was removed, and the air-dried coverslips were rehydrated in PBS and stained with 100 nM SYTOXgreen for 30 min in the dark at room temperature. After washing three times with nuclease free water (Sigma-Aldrich), the samples were mounted with ProLong Gold antifade reagent (Invitrogen) and analyzed under an Axioskop 40 fluorescence microscope using the Axio Vision Rel. 4.8-Software (both Carl Zeiss, Jena, Germany). For staining of neutrophil elastase, after rehydration with PBS, samples were blocked overnight with 10% normal goat serum (Jackson Immuno Research, Newmarket, UK), 5% cold water fish gelatine (Sigma-Aldrich), 1% bovine serum albumin (Roth, Karlsruhe, Germany), and 0.05% Tween 20 (Serva, Heidelberg, Germany) diluted in PBS. Afterwards the samples were washed with PBS and incubated with anti-neutrophil elastase rabbit pAb (1 : 200; Calbiochem, Merck, Darmstadt, Germany) for 1 h at 37°C. After washing three times with PBS, the primary antibody was detected with Cy3-AffiniPure anti-Rabbit IgG (1 : 100; Jackson Immuno Research, Newmarket, UK) for 1 h and DNA was stained with 100 nM SYTOXgreen. Samples for scanning electron microscopy were fixed in a Monti-Graziadei solution (2% glutaraldehyde, 0.6% paraformaldehyde in 0.1 M cacodylate buffer, at pH 7.2) for 2 days and then dehydrated in a rising alcohol series (30, 40, 50, 60, 70, 80, 90, and 100% for 15 min each). After critical-point drying, the samples were placed on aluminium slides and subsequently sputtered with gold. Samples were examined in a SEM 505 (Philips, Eindhoven, Holland).

### 2.7. Statistical Analysis

Statistical analysis was performed with graph pad prism 4 software. Data were analyzed using the one way Anova test and Bonferroni posttest.

## 3. Results and Discussion

### 3.1. The Applied Inhibitors of ROS Production Are Not Toxic and Do Not Induce Apoptosis in Neutrophils

**Figure 2:**
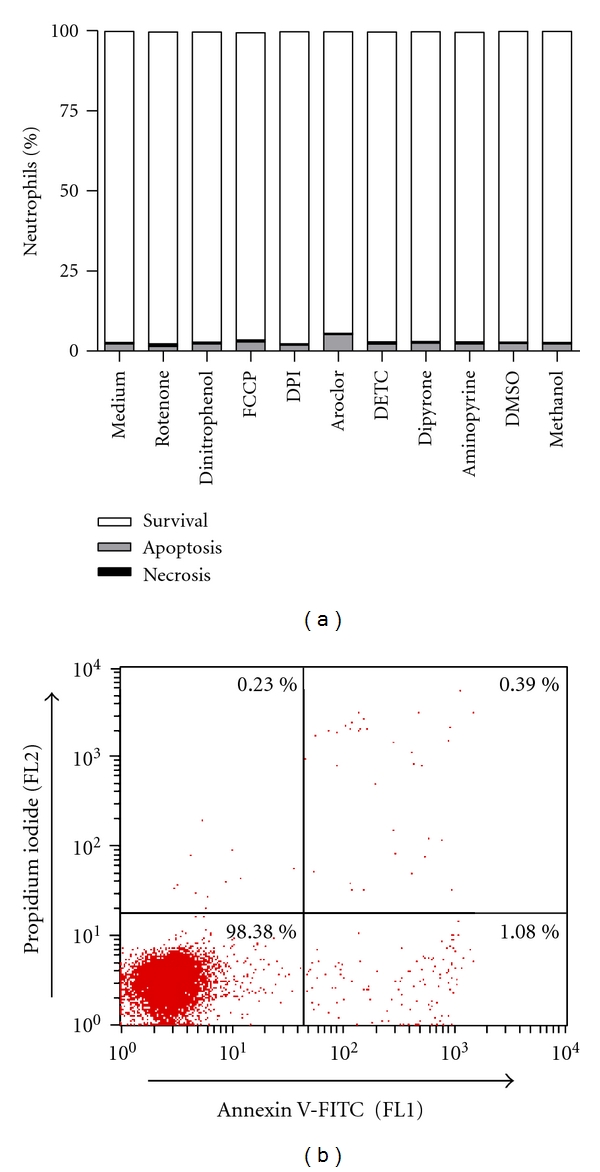
Inhibitors of various steps of ROS production are not toxic and do not induce apoptosis in human neutrophils. Freshly isolated human neutrophils were incubated with or without inhibitors (10 *μ*M Rotenone, 10 *μ*M Dinitrophenol, 500 nM FCCP, 20 *μ*M DPI, 38 *μ*M Aroclor, 10 *μ*M DETC, 200 *μ*M Dipyrone, and 200 *μ*M Aminopyrine) for 4 h at 37°C. DMSO and methanol were used as solvent controls for Rotenone and Aroclor. Apoptosis and cell death were assessed by flow cytometry using stainings with Annexin V-FITC and propidium iodide, respectively. (a) The effect of various inhibitors on apoptosis and necrosis of neutrophils *in vitro*. Data are shown as mean from 3 independent experiments. (b) Representative dot plot of a flow cytometry analysis of human neutrophils cultured without inhibitor. The *x*-axis shows the Annexin V-FITC fluorescence, at the *y*-axis the propidium iodide fluorescence is indicated. Events in the lower right quadrante (1.08%) represent the apoptotic cells whereas events in the upper quadrants represent the necrotic population.

For the inhibitors acting on various steps of intracellular ROS production (Figure 1) effective concentrations were chosen either based on previous reports [5, 15–19] or by our own preliminary studies (data not shown). To exclude a toxic effect of the inhibitors, all inhibitors were tested for their toxicity (necrosis) and apoptosis-inducing effect on primary human neutrophils. By using flow cytometry analysis of a double staining with Annexin V-FITC and propidium iodide, none of the applied inhibitors exerted a toxic or apoptosis-inducing effect after 4 h (Figure 2). Necrosis or apoptosis induction was also not seen after longer (18 h) incubation (data not shown).

### 3.2. The Effect of Various Inhibitors on the Intra- and/or Extracellular ROS Production by Neutrophils

**Figure 3:**
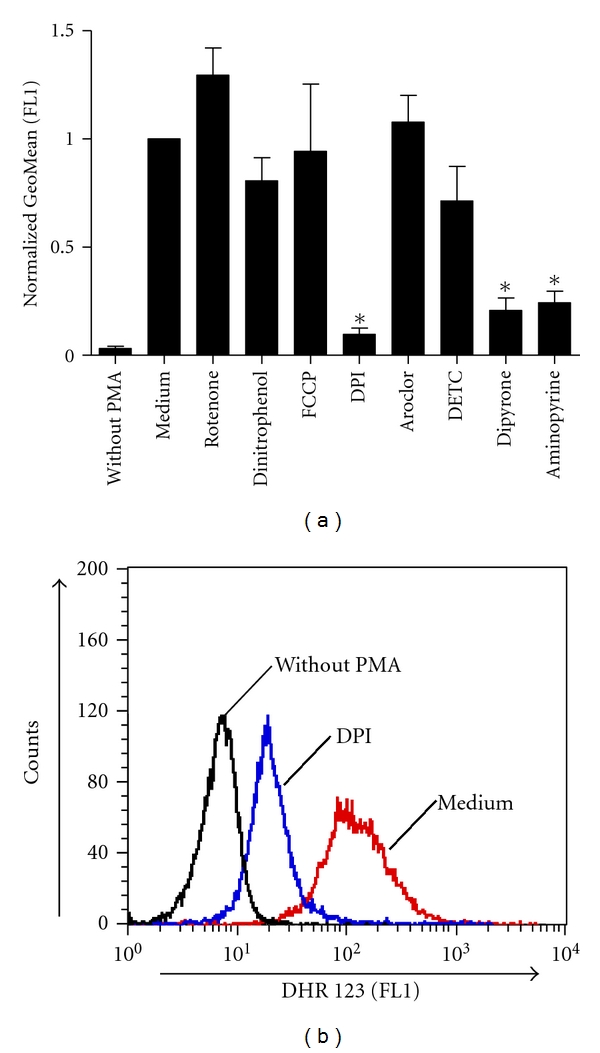
Flow cytometry analysis of intracellular ROS production by neutrophils after exposure to various inhibitors. Freshly isolated human neutrophils were incubated with or without inhibitors (10 *μ*M Rotenone, 10 *μ*M Dinitrophenol, 500 nM FCCP, 20 *μ*M DPI, 38 *μ*M Aroclor, 10 *μ*M DETC, 200 *μ*M Dipyrone, and 200 *μ*M Aminopyrine) for 30 min at 37°C. Cells were loaded with DHR 123, and the samples were stimulated with 4 *μ*M PMA. The intracellular production of ROS was assessed by using flow cytometry. (a) Geometrical mean (GeoMean) values of the fluorescence intensities normalized to the PMA-induced ROS production in the absence of inhibitors (Medium). Data show mean ± SD from four independent experiments, *: *P* < 0.001 as compared to the PMA-stimulated sample without inhibitor (Medium). (b) Representative histogram showing the fluorescent intensities of unstimulated neutrophils (without PMA), PMA-stimulated neutrophils without inhibitor (Medium), and, as an example for an inhibitory effect, PMA-stimulated neutrophils after exposure to DPI. Data are from one experiment representative for three independent experiments.

Prior to testing their effects on NET release, all inhibitors were tested for their efficacy to inhibit PMA-induced ROS production. Three widely used ROS detection methods were tested for their applicability for our project. As a first approach, the intracellular ROS production in neutrophils loaded with DHR 123 was measured by using flow cytometry. H_2_O_2_ rather than O_2_^∙−^ is required to oxidise the nonfluorescent DHR 123 to the fluorescent Rhodamin 123 derivate [20]. This slow reaction is catalysed by enzymes with peroxidase activity such as MPO. Although the DHR oxidation is promoted by H_2_O_2_, this assay can also be used to detect O_2_^∙−^, because the dismutation of O_2_^∙−^ to H_2_O_2_ and oxygen is rapid even in the absence of SOD [20]. DPI, an inhibitor of NOX and complex I of the mitochondrial electron transport chain, exerted a strong inhibitory effect on intracellular ROS production measured in this assay (Figure 3). The SOD inhibitors Aroclor and DETC had no inhibitory effect. The MPO inhibitors, Dipyrone and Aminopyrine, also decreased the fluorescence intensity (Figure 3(a)).

Although the DHR 123-based technique is simple and rapid, this method is not very sensitive [21]. This could possibly be the reason why high PMA concentrations were needed to detect a burst and no clear results were obtained for the mitochondrial inhibitors (Figure 3(a)). In subsequent experiments more sensitive test methods were applied.

**Figure 4:**
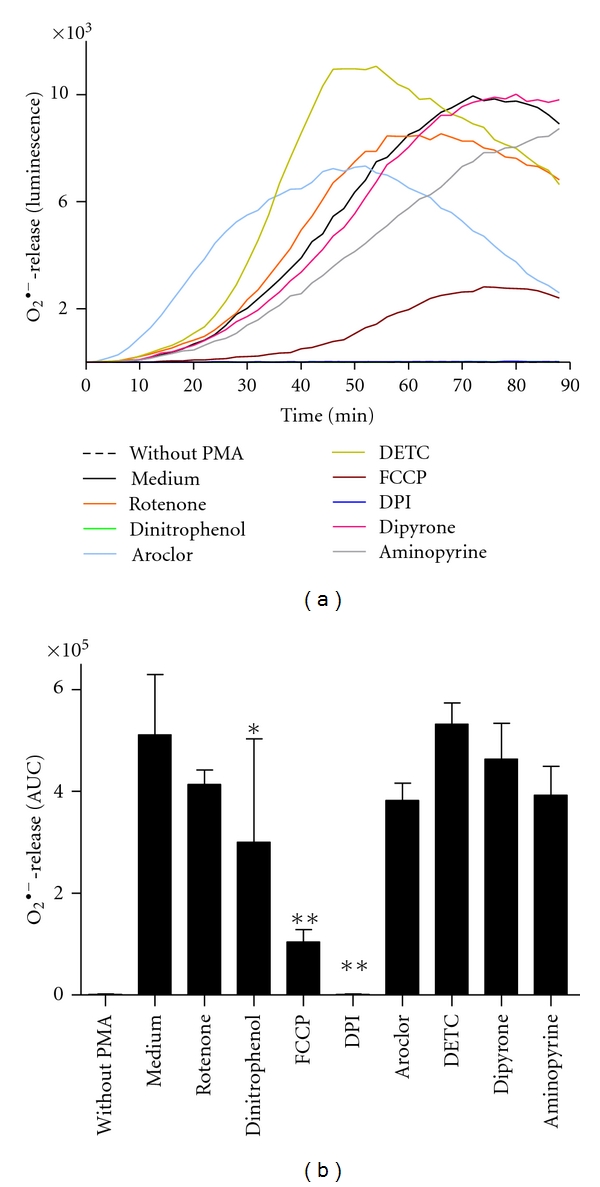
Extracellular ROS production of neutrophils incubated with various inhibitors as measured by the lucigenin-amplified chemiluminescence assay. Freshly isolated human neutrophils were incubated with or without inhibitors (10 *μ*M Rotenone, 10 *μ*M Dinitrophenol, 500 nM FCCP, 20 *μ*M DPI, 38 *μ*M Aroclor, 10 *μ*M DETC, 200 *μ*M Dipyrone, and 200 *μ*M Aminopyrine) for 30 min at 37°C. Subsequently, lucigenin was added, and the ROS production was induced with 20 nM PMA. The O_2_^∙−^-release was detected by measuring chemiluminescence for a period of 1 h at 37°C. The control without PMA shows the O_2_^∙−^-release of unstimulated neutrophils. (a) Time kinetics of O_2_^∙−^-release (chemiluminescence) in one representative experiment. (b) O_2_^∙−^-release quantified by calculation of the area under the curve (AUC). Data show mean ± SD from four independent experiments, **: *P* < 0.001; *: *P* < 0.01 as compared to the PMA-stimulated sample without inhibitor (Medium).

The lucigenin-amplified chemiluminescence assay is a sensitive technique to quantify extracellular ROS, mainly superoxide anions (O_2_^∙−^) [12, 13]. By using this technique a strong inhibitory effect was observed for DPI (Figure 4), which completely abolishes superoxide production. In addition, the uncoupling mitochondrial chain reagents FCCP and Dinitrophenol exerted significant inhibitory effects (Figure 4(b)). The other inhibitors did not exert a marked effect by using the lucigenin-amplified chemiluminescence assay (Figure 4).

**Figure 5:**
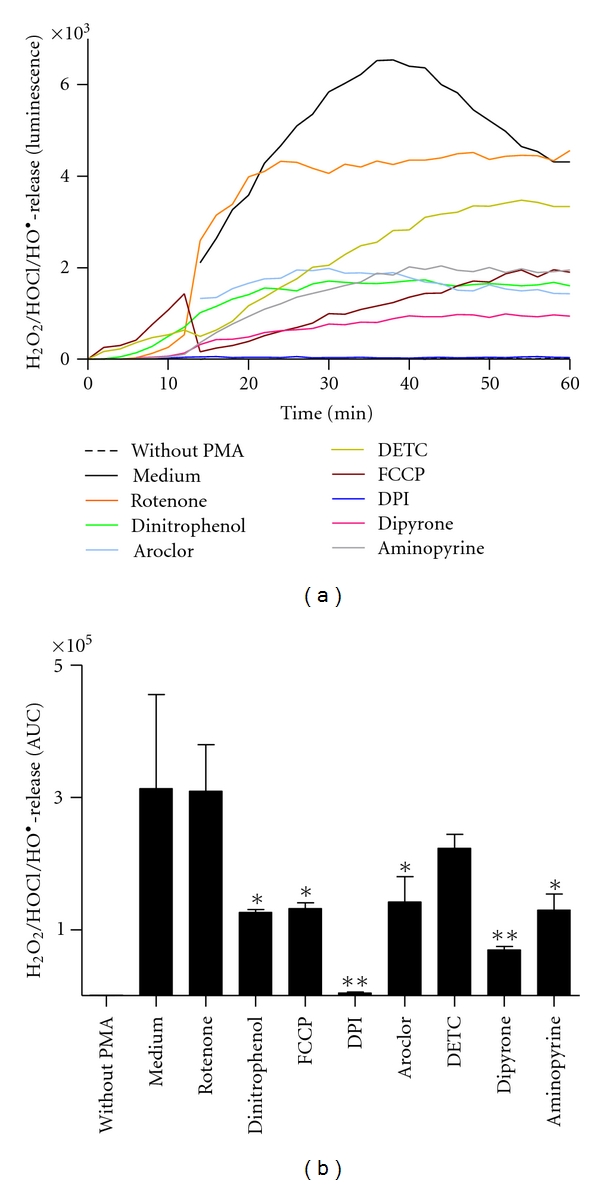
Assessment of intra- and extracellular ROS production of neutrophils after exposure to various inhibitors as measured by the luminol-amplified chemiluminescence assay. Freshly isolated human neutrophils were incubated with or without inhibitors (10 *μ*M Rotenone, 10 *μ*M Dinitrophenol, 500 nM FCCP, 20 *μ*M DPI, 38 *μ*M Aroclor, 10 *μ*M DETC, 200 *μ*M Dipyrone, and 200 *μ*M Aminopyrine) for 30 min at 37°C. Subsequently, luminol was added, and the ROS production was induced with 20 nM PMA. The H_2_O_2_ release was detected by measuring chemiluminescence for a period of 1 h at 37°C. (a) Time kinetics of H_2_O_2_ release (chemiluminescence) of one representative experiment. (b) H_2_O_2_ release quantified by calculation of the area under the curve (AUC). Data show mean ± SD from three independent experiments, **: *P* < 0.001; *: *P* < 0.01 as compared to the PMA-stimulated sample without inhibitor (Medium).

As a third technique the luminol-amplified chemiluminescence assay was applied to assess the effects of various inhibitors on ROS production. This technique detects both intra- and extracellular ROS. In contrast to lucigenin, which is mainly oxidized by O_2_^∙−^, luminol can be oxidized by several ROS, such as H_2_O_2_, HOCl, and HO^•^ [22, 23]. Experiments using this technique revealed that DPI nearly completely abolished ROS production (Figure 5). The MPO-inhibitors Dipyrone and Aminopyrine prevented the MPO-induced oxidation of luminol with high efficiency (Figure 5), but had no inhibitory effect in the lucigenin assay (Figure 4), indicating that Aminopyrine und Dipyrone have no inhibitory effect on NOX. The mitochondrial uncoupling reagents FCCP and Dinitrophenol exerted significant inhibitory effects as well (Figure 5(b)). Rotenone, however, did not inhibit PMA-induced ROS production. From the two tested SOD inhibitors Aroclor exerted a significant inhibitory effect. The effect of DETC was statistically not significant (Figure 5(b)).

### 3.3. The Effect of Various Inhibitors of ROS Production on NET Release by Primary Human Neutrophils

Major sources of ROS in neutrophils are the enzymatic reactions mediated by NADPH oxidase and MPO [19, 24]. In addition, ROS are produced in mitochondria through the function of the mitochondrial electron transport chain. Since PMA-induced NET release was shown to depend on ROS [2] it was to expect that inhibition of pathways involved in ROS generation leads to reduction of NET formation. The above-described assays confirmed the inhibitory effect of DPI, Dipyrone, Aminopyrine, FCCP, Dinitrophenol, and Aroclor on the ROS production and in part on ROS release by human neutrophils.

**Figure 6:**
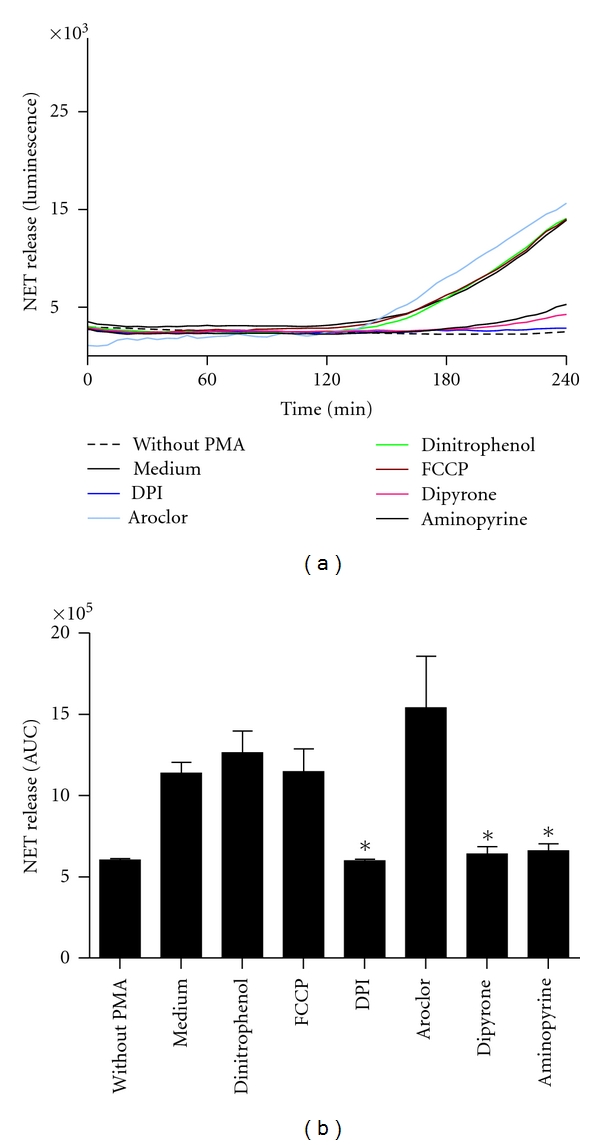
Release of NETs by neutrophils after exposure to various ROS inhibitors as measured by the SYTOXgreen assay. Freshly isolated human neutrophils were incubated with or without inhibitors (10 *μ*M Dinitrophenol, 500 nM FCCP, 20 *μ*M DPI, 38 *μ*M Aroclor, 200 *μ*M Dipyrone, and 200 *μ*M Aminopyrine) for 30 min at 37°C. Subsequently, 5 *μ*M SYTOXgreen was added to all samples, and formation of NETs was induced by 20 nM PMA. Samples without PMA were used as control. The NET production was detected for a period of 4 h at 37°C by measuring the fluorescence of DNA-bound SYTOXgreen. (a) Time kinetics of NET release (fluorescence intensity) of one representative experiment. (b) Quantification of NET release by calculation of the area under the curve (AUC). Data show mean ± SD from three independent experiments, *: *P* < 0.01 as compared to the PMA-stimulated sample without inhibitor (Medium).

The effect of these inhibitors on the PMA-induced NET release by neutrophils was assessed. In these experiments only those substances were tested that showed an inhibitory effect on ROS production in any of the above-described ROS production tests. The panel of inhibitors contained substances targeting the NOX-mediated and/or mitochondrial ROS production, as well as SOD- or MPO-derived ROS (Figure 1). Human neutrophils were preincubated for 30 min with the inhibitors prior to NET induction with PMA. The NET release was quantified by measuring the fluorescence of SYTOXgreen over a period of 4 h. Inhibition of NADPH oxidase with the inhibitor DPI abolished NET release (Figure 6). DPI, however, in addition to NADPH oxidase inhibits also the mitochondrial electron transport chain [25]. Therefore, the observed NET-inhibiting effect of DPI can be a result of its known inhibitory effect on NADPH oxidase or on mitochondrial ROS production or both. In order to dissect the two possible targets of DPI, experiments were carried out with inhibitors targeting the mitochondrial ROS release. The studies using mitochondrial inhibitors aimed also to decipher the contribution of mitochondrial ROS production on the NET formation. To inhibit mitochondrial ROS production, we used FCCP and Dinitrophenol, two substances that uncouple the mitochondrial oxidative phosphorylation. Uncoupler agents dissipate the mitochondrial membrane potential which is generated for the production of ATP by transporting protons across the membrane. Consequently, the electron transport chain functions more efficiently to reestablish the proton gradient that results into less leakage of electrons, and therefore less ROS are generated [26–28]. Both FCCP and Dinitrophenol were shown previously to inhibit the production of ROS in PMA-stimulated neutrophils [29, 30]. In addition, the FCCP-mediated reduction in ROS release is due to the mitochondrial uncoupling [29]. An unspecific inhibition of oxidative burst could be excluded at concentrations below 1 *μ*M [29]. Although the mitochondrial inhibitors FCCP and Dinitrophenol significantly reduced ROS generation in neutrophils (Figures 4(b) and 5(b)), no inhibitory effect on NET release was observed (Figure 6). These findings indicate that mitochondrial ROS do not play a major role in the PMA-induced NET release. Our experiments with the mitochondrial inhibitors also indicate that the inhibitory effect of DPI on NET release is associated with the effect of DPI on NADPH oxidase.

As described previously, MPO is required for PMA-induced NET release [6]. To further elucidate the role of MPO-derived ROS, experiments with Aminopyrine and Dipyrone were carried out to assess the contribution of MPO to the NET release. It has been shown previously that these inhibitors prevent PMA-induced ROS production in human neutrophils [19]. This effect is due to the scavenging of hypochlorite and hydroxyl radicals by Aminopyrine and Dipyrone [19] and to the inhibition of MPO by Aminopyrine [31]. Our data show that Aminopyrine and Dipyrone prevent the MPO-induced oxidation of DHR 123 and luminol (Figures 3 and 5) but failed to inhibit the superoxide-dependent oxidation of lucigenin (Figure 4). In line with previous findings [19], these results indicate that Aminopyrine and Dipyrone have no scavenging effect against superoxide and no inhibitory effect on NOX. Both Dipyrone and Aminopyrine inhibited significantly NET release (Figure 6(b)). These results provide experimental evidence that MPO enzymatic activity and MPO-derived ROS are required for NET release. Hypochlorite (OCl^−^) is the likely MPO-derived ROS involved in the process of NET formation. The interaction of hypochlorite with hydrogen peroxide (H_2_O_2_) leads to the generation of singlet oxygen. Singlet oxygen has been reported to be essential for NET formation [8].

As inhibitor of SOD we used Aroclor, a mixture of polychlorinated biphenyls. In a previous study it was shown that preincubation of human neutrophils with Aroclor impaired the PMA-induced ROS release by inhibiting SOD, which converts O_2_^∙−^ to H_2_O_2_ and oxygen [17]. In line with this finding we observed a significant downregulation of H_2_O_2_ by using the luminol assay (Figure 5). Exposure of neutrophils to the SOD inhibitor Aroclor did not significantly affect PMA-induced NET release (Figure 6). Apparently this finding contradicts the observations regarding the involvement of MPO-derived ROS and singlet oxygen [8] in NET release. However, in a previous study SOD was also not required for PMA-induced hypochlorite (OCl^−^) production [32]. SOD appears to be not essential for the production of hydrogen peroxide (H_2_O_2_). Indeed superoxide can be dismutated to hydrogen peroxide spontaneously, without the need for enzymatic dismutase activity [33]. This spontaneous dismutase reaction increases with decreasing pH value and can convert significant levels of superoxide to hydrogen peroxide at neutral pH with a rate of 2 × 10^5^ M^−1^s^−1^ [33]. Interestingly, although hydrogen peroxide release was significantly downregulated by Aroclor (Figure 5), Aroclor had no significant effect on the NET release. Neutrophils from some of the donors, however, exhibited an increased NET formation (Figure 6(a)). This could be due to the fact that inhibition of SOD leads to an imbalance between production of free radicals and antioxidant defense mechanism. It has been shown previously that in parallel to a decrease in H_2_O_2_ production, O_2_^∙−^ production increases by use of Aroclor [17]. We observed a minor, but statistically not significant Aroclor-induced increase in superoxide production by using the DHR 123 assay (Figure 3). This effect could be responsible for the slightly enhanced NET release observed in some of the experiments.

**Figure 7:**
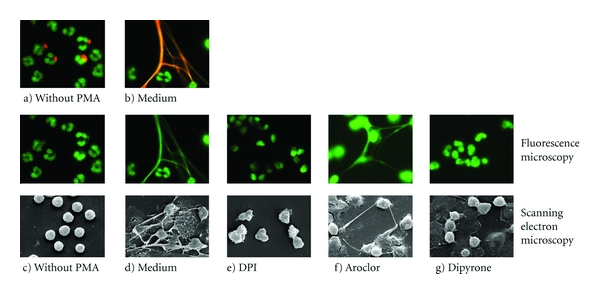
Release of NETs by neutrophils after exposure to various ROS inhibitors assessed by fluorescent and scanning electron microscopy (SEM). Freshly isolated human neutrophils were preincubated with various ROS inhibitors (20 *μ*M DPI, 38 *μ*M Aroclor, and 200 *μ*M Dipyrone) for 30 min at 37°C. NET release was induced by 20 nM PMA, and the samples were fixed after 4 h. NET release was detected by fluorescent microscopy and SEM. (a-b) DNA staining with SYTOXgreen and neutrophil elastase or (c–g) with SYTOXgreen alone. (a) and (c) unstimulated neutrophils, (b) and (d) PMA-stimulated neutrophils in medium (without inhibitor), (e–g) PMA-stimulated neutrophils exposed to a ROS inhibitor.

Fluorescent microscopy and scanning electron microscopy (SEM) were used to verify the effects observed by using the quantitative NET release technique. NET formation was visualized at 4 h after PMA stimulation. As examples for the effect of various inhibitors, Figures 7(e) and 7(g) confirm the absence of NETs in the presence of DPI and Dipyrone, respectively. The microscopical analysis also confirmed that the SOD-inhibitor Aroclor does not inhibit PMA-induced NET release (Figure 7(f)).

The release of NETs is an important antimicrobial effector mechanism of neutrophils. However, NETs can be harmful if they are released in the absence of microbial infections, such as in autoinflammatory or autoimmune disorders. A dysregulation of NETs has been shown, for example, in small-vessel vasculitis [34] and systemic lupus erythematosus [35]. Increased knowledge of the mechanisms involved in NET release would lead to better understanding regarding the pathophysiology of autoinflammatory and autoimmune diseases and may lead to the development of novel therapeutic strategies. Our study indicates that NOX- and MPO-derived ROS, but not mitochondrial ROS are important for the release of NETs. In addition, we could show that ROS scavengers such as Aminopyrine and Dipyrone effectively inhibit NET release. Therefore, ROS scavengers could be potential therapeutic agents for the suppression of NET release.

## 4. Conclusions

The presented data indicate the importance of NADPH oxidase- and myeloperoxidase-(MPO-) derived ROS for the PMA-induced formation of neutrophil extracellular traps (NETs). Mitochondria-derived ROS as well as the contribution of superoxide dismutase (SOD) are, however, not essential for the release of NETs.
